# A New Look at Some Old Animals

**DOI:** 10.1371/journal.pbio.1000007

**Published:** 2009-01-27

**Authors:** Neil W Blackstone

## Abstract

How the tiny marine animal*Trichoplax adhaerens* is related to other animals has long puzzled researchers studying the origin of metazoans. An ambitious "total evidence" study provides careful analysis of this question and reveals some surprises.

When teaching an introductory zoology course, it is always entertaining to show students some specimens of the only described species of placozoans—Trichoplax adhaerens, a tiny, simple, nearly worldwide marine organism (Figure 1) [1,2]. The inevitable disbelief—those are animals?—leads naturally to enumerating the shared derived features of all animals (Box 1) and in turn to discussing the relationships among the various groups at the base of the animal or “metazoan” tree of life. These early diverging or “basal” groups include bilaterians, the bilaterally symmetric forms that most students recognize as animals—worms, flies, mice, and many more. Most students are also familiar with cnidarians—corals, anemones, jellyfish, hydroids—and perhaps even with sponges, known for their soft and porous skeletons. Less familiar are the ctenophores—comb jellyfish—and the enigmatic placozoans. How these five groups fit together at the root of the metazoan tree is a matter of intense debate and considerable study. Indeed, the ordering of the divergence of these basal groups affects our inferences of the features of the common ancestor of all animals. This in turn influences our understanding of the evolution of all animal characteristics, whether molecular, physiological, or morphological.

**Figure 1:**
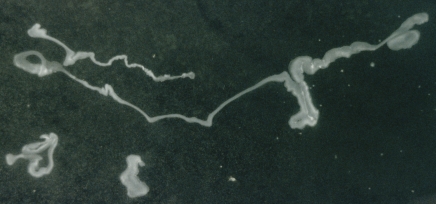
Trichoplax adhaerens Individuals Creeping along the Glass Wall of an Aquarium The nearly “rounded up” individual in the lower center of the image is several millimeters long. Extremely elongate forms such as these are often seen in large aggregations of Trichoplax individuals.

**Box 1. Characteristics of Animals**

Animals or “metazoans” are typically heterotrophic, multicellular organisms with diploid, eukaryotic cells. They are defined by a number of features, including several related to gamete formation and structure [3]. Since the life cycle of Trichoplax is incompletely known, these characteristics are of little use in this context. Presence of a collagenous extracellular matrix is often used to define animals; in the case of Trichoplax, the absence of such a matrix would then have to be interpreted as a secondary loss [3]. Animals, however, are also defined by the presence of different somatic (i.e., non-reproductive) cell types and by impermeable cell–cell connections. By these criteria, Trichoplax are animals, while related multicellular protists (e.g., choanoflagellates) are not.

## Placozoans and the Root of the Metazoan Tree

Of the five groups, the position of the placozoans has perhaps been the most contentious. They are clearly animals by virtue of having four somatic (i.e., non-reproductive) cell types—cover, cylinder, gland, and fiber cells [1,2]. All other animals, however, have many more somatic cell types. Further, the cell-level dynamics of Trichoplax are unusual. While cylinder cells may give rise to gland cells, otherwise the three major cell types (cover, cylinder, and fiber) give rise to their own cell type and none other during growth and reproduction. In contrast to other early diverging animals, placozoans do not seem to have a stem cell lineage that gives rise to more than one cell type (but see [2] for further discussion). Although the process is incompletely studied, placozoans do form germ cells, apparently from the somatic cells of the lower epithelium [1]. Cells are organized into two surface layers—a functional lower and upper side. Both cell layers lack underlying “basal lamina”—an extracellular matrix on which the cells sit—or other traces of such a matrix. These microscopic structures are found in all other animals [1–3]. Both sides of a placozoan are covered with flagella, with a higher density on the lower side. Morphologically, a living Trichoplax resembles a small, often highly irregular “plate” of cells, 2–3 mm in diameter, moving by means of flagella and constantly changing in outline (Video S1). Individuals are free-living and heterotrophic, but their natural history remains poorly known [4].

When considering such a creature, biologists must try to determine whether the observed simplicity is primary or secondary. In other words, was the evolutionary lineage leading to Trichoplax always highly simplified, or is Trichoplax the simplified descendent of a more complex ancestor? The latter situation is commonly found in many parasitic species but is considerably less common in free-living ones. In the late 19th century, the first descriptions of Trichoplax suggested that it exhibited primary simplicity [2]. This view was enthusiastically incorporated into “scenario-based” views of animal evolution, in which biological observations are synthesized into plausible historical narratives. In particular Otto Bütschli developed the “placula hypothesis,” which featured a Trichoplax-like organism as the ancestor of all animals [2]. As with other animals, of course, modern placozoans are separated from such an ancestor by perhaps a billion years of evolution. Many features of modern Trichoplax may thus differ from such a putative ancestor.

**Figure 2:**
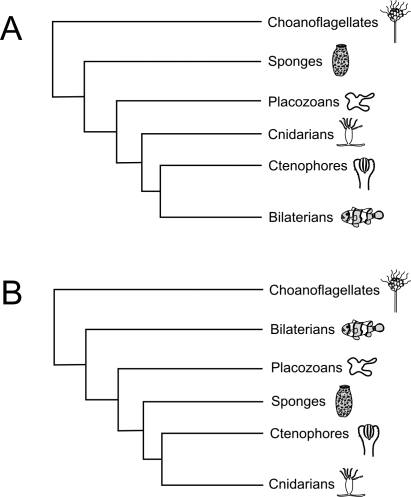
Schemata of Two Hypotheses for the Branching Order of Groups at the Root of the Metazoan Tree (A) One of several competing hypotheses for early metazoan evolution: the choanoflagellates (which are not animals; see Box 1) serve as an outgroup in the analysis, and sponges are the sister group to the placozoan + cnidarian + ctenophore + bilaterian clade (see [6]). (B) A simplified view of the hypothesis of Schierwater et al. [10]: bilaterians are the sister group to the placozoan + sponge + ctenophore + cnidarian clade, while placozoans are the sister group to the sponge + ctenophore + cnidarian clade. (Artwork courtesy of Austin Parrin)

By the early 20th century, however, the view of Trichoplax as secondarily simplified became widely accepted. For some time, placozoans were classified as degenerate cnidarian larvae (see [5] for discussion). While careful study of the morphology in fact provides little support for this notion [3,5], in the case of such divergent opinions it is often helpful to look at other sources of information. Indeed, by the late 20th century DNA sequence data became widely available. Such data are particularly helpful with simple organisms such as Trichoplax, which exhibit relatively few morphological characters. Also by this time, considerably more rigorous methods had been developed for evaluating phylogenetic hypotheses. Based on a large number of studies [6], such data generally did not support the view that placozoans were simplified cnidarians. At least in broad outline, fairly well-supported hypotheses of the metazoan tree of life began to take shape (e.g., Figure 2A).

In this context, the mitochondrial genome of Trichoplax provided some surprises. While animal mitochondrial genomes are relatively stereotypical in terms of size and gene content, the Trichoplax genome was more than twice as large and contained unusual protein-coding regions [7]. Mitochondria are descendents of symbiotic bacteria and have moved most of their genes to the nucleus [8]. This evidence thus appeared particularly strong: placozoans had diverged from the lineage leading to other animals before large segments of mitochondrial DNA had moved to the nucleus. On the other hand, analyses of nuclear genes [9] supported the alternative view with the placement of sponges as sister to the placozoan + cnidarian + bilaterian clade (e.g., Figure 2A). In this case, the similarities between sponge mitochondrial genomes on one hand and cnidarian + bilaterian mitochondrial genomes on the other are viewed as a case of parallel evolution or “parallelism,” in which the same underlying evolutionary process (i.e., movement of mitochondrial genes to the nucleus) occurs in different lineages to produce similar character states.

## Concatenated Molecular and Morphological Analysis

Could a simultaneous analysis of morphology, mitochondrial DNA, nuclear DNA, and other available characters reconcile these divergent views? Such an approach is taken by Schierwater and colleagues in a new *PLoS Biology* study [10]. The rationale is clear—there can only be one phylogeny for the five animal groups in question. Nevertheless, this analysis is far from straightforward. Not only is there an enormous volume of data, but in some cases it is difficult to include different sorts of data in the same analysis. For instance, one kind of data may swamp out the signal from another data set by sheer abundance. In addition, if the analysis being done relies on a model of evolution, the particular model may not apply to different sorts of data. Further, given that mitochondrial and nuclear data support different conclusions [7,9], how can it be determined if the final tree is largely a product of the signal from one data set or the other? These and a number of other issues are carefully considered by Schierwater and colleagues [10] and elsewhere [11]. This approach provides rigorous taxon sampling, the most inclusive data set, and the most comprehensive tree building analyses available so far. While debates about methodological issues will no doubt continue, the results of the analysis represent a striking departure from some widely accepted views of the animal tree of life (Figure 2B).

In the best-supported trees, placozoans are sister to the sponge + ctenophore + cnidarian clade. In view of such results, it is interesting to reconsider the placula hypothesis. While speculative, this hypothesis nevertheless provides a useful framework for organizing information and testing hypotheses. As an exemplar, the authors examine the spatial expression of genes that regulate pattern formation and point out interesting congruence with the placula hypothesis. In addition to the placement of the placozoans, the results provide a broader reinterpretation of animal relationships. In particular, the result that will receive the most comment is the position of the bilaterians as sister to the placozoan + sponge + ctenophore + cnidarian clade (Figure 2B). This is in sharp contrast to hypotheses in which bilaterians nest within these other groups (e.g., Figure 2A). Indeed, the common views of “higher” and “lower” animals follow from such nesting. Evolution, however, need not be progressive. Certainly, a broad literature supports the notion that there are enormous differences between bilaterians and these other early evolving animals. Considerations of regulatory gene evolution [12], the evolution of the germ line [13–15], and patterns of development and aging [16,17] all suggest a wide gulf between bilaterians and other basal groups. Could there be two basic kinds of animals, represented by bilaterians on the one hand and by placozoans, sponges, cnidarians, and ctenophores on the other? As the authors note [10], such a view would require considerable parallelism beyond that discussed above with regard to the mitochondrial genome, e.g., in the evolution of the nervous system. Nevertheless, a number of other studies suggest that parallelism is a prominent feature of metazoan evolution [18,19]. Such intriguing questions will no doubt stimulate considerable amounts of additional research on the relationship between these five early evolving animal groups. While Schierwater and colleagues have set a new methodological standard for subsequent studies, their results also suggest a gap in our current knowledge: we need a clearer picture of the base of the bilaterian tree to fully understand animal evolution [14].

## Supporting Information

Video S1 Trichoplax in Motion A Trichoplax individual, roughly 2–3 mm in diameter, detaches from the substratum, curls up into a tube, and prepares to drift or swim away. Swimming and drifting are apparently common in Trichoplax in the field [4].
(2.9 MB WMV).
